# Does the Simultaneous Introduction of Several Pharmaceuticals in the Post-Lenalidomide Era Translate to Better Outcomes in Relapse Refractory Multiple Myeloma? Findings from the Real-World Innovation in Multiple Myeloma (REAL IMM) Study

**DOI:** 10.3390/cancers15245846

**Published:** 2023-12-14

**Authors:** Ioannis Petrakis, Christos Kontogiorgis, Evangelia Nena, Sosana Delimpasi, Natasa E. Loutsidi, Emmanouil Spanoudakis, Stergios Intzes, Christina Misidou, Marianthi Symeonidou, Nikolaos Giannakoulas, Theodoros C. Constantinidis, Evangelos Terpos

**Affiliations:** 1 Laboratory of Hygiene and Environmental Protection, School of Medicine, Democritus University of Thrace, 68100 Alexandroupolis, Greece; ckontogi@med.duth.gr (C.K.); tconstan@med.duth.gr (T.C.C.); 2 Bone Marrow Transplantation Unit, Department of Hematology, Evangelismos Hospital, 10676 Athens, Greece; sodeli@yahoo.com (S.D.); natloutsidi@gmail.com (N.E.L.); 3 Department of Hematology, School of Medicine, Democritus University of Thrace, 68100 Alexandroupolis, Greece; espanoud@med.duth.gr (E.S.); instergios@hotmail.com (S.I.); xmisidou@yahoo.gr (C.M.); marianthi.symeonidou@hotmail.com (M.S.); 4 Department of Hematology, School of Medicine, University of Thessaly, 41110 Larisa, Greece; ngiannak@uth.gr; 5 Department of Clinical Therapeutics, School of Medicine, National and Kapodistrian University of Athens, 11528 Athens, Greece; eterpos@med.uoa.gr

**Keywords:** carfilzomib, daratumumab, ixazomib, pomalidomide, multiple myeloma, real world

## Abstract

**Simple Summary:**

On rare occasions, a handful of innovative cancer drugs may successfully go through clinical trials and manage to obtain simultaneous regulatory approvals to treat a specific disease, providing hope for improved treatment outcomes. In the past few years, physicians in North America, Europe, and other countries with stronger economies have been able to use several of the recently approved drugs to treat a blood cancer called multiple myeloma, once older therapies have failed. This study looked at the treatment outcomes associated with the wave of newer drugs in patients diagnosed with multiple myeloma in Greece. The results showed that approximately three-quarters of patients who received one of the newer drugs after having progressed on front-line therapies remained in remission a year after initiating treatment. This finding was comparable to the results from previous studies in Greek patients who were diagnosed with relapsed or refractory multiple myeloma and had previously used other therapies.

**Abstract:**

Newer methodologies are needed to assess the real-world comparative effectiveness of a “generation” of pharmaceutical innovation versus the prior standard of care. This chart review study aimed to first evaluate the cumulative clinical benefits of pharmaceutical innovation in everyday relapse/refractory multiple myeloma before analyzing findings in the context of respective real-world outcomes from the bortezomib/lenalidomide era. Study endpoints included the 52-week PFS rate in second and third line of therapy (LOT), mPFS-2 across the first and second LOT, the ORR, reasons for discontinuation, and the treatment duration per therapeutic algorithm. Data from 107 patients were collected. The median follow-up was 2.0 years. Of the subjects who met the selection criteria for the second LOT, 72.2% maintained the PFS at 52 weeks. In the third-line setting, the PFS rate at 52 weeks was 63.5%. The mPFS across the first and second, the second, and the third LOTs were 26, 17, and 15 months, respectively. The ORR was 76.1% in the second and 69.7% in the third LOT. After non-response or progression, the main reason for drug discontinuation was treatment intolerability. The second-line ORR and the 52-week PFS rate were similar to previous real-world findings from the bortezomib/lenalidomide era. The cumulative mPFS across the second and third LOTs was higher than the respective mPFS across the first and second LOTs. Despite its limitations, the methodology and findings from this study may be used in future clinical and economic evaluations across all hematological malignancies.

## 1. Introduction

Multiple myeloma (MM) is characterized by the presence of abnormal clonal plasma cells in the bone marrow, often leading to uncontrolled growth causing destructive bone lesions, among other signs and symptoms [1]. There has been an increasing trend in the global MM incidence, with an age-standardized rate of 1.78 per 100,000 people estimated in 2020 [2]. MM is the second-most common hematological malignancy and is still considered an incurable cancer; however, such a fundamental dogma is hopefully fading due to novel therapies, optimized diagnosis, and improved transplantation strategies [3,4,5].

Relapse refractory multiple myeloma (RRMM), with or without prior autologous stem cell transplantation, carries a significant unmet need for patients and physicians [6]. Managing relapse can be complex not only in terms of personalized treatment needs but also due to tolerability challenges with some of the newer drug combinations [7]. Several novel agents, including carfilzomib, daratumumab, elotuzumab, ixazomib, pomalidomide, and panobinostat, were approved around 2015 [8]. Almost 10 years later, the residual high unmet need coupled with the industry’s strategic interest expedited innovation and surfaced new evidence supporting international clinical guideline updates [9], regulatory label expansions across a multitude of combination therapies, and also newer approvals for novel agents, including several immunotherapy modalities [10].

To assess the benefits associated with the most recent wave of innovation in RRMM, the hemato-oncology and healthcare policy communities aim to better understand the retrospective pharmacoepidemiological and pharmacoeconomic footprints of historical approvals in the post-bortezomib and post-lenalidomide era. Despite the disproportional recent advancements of pharmaceutical innovation in MM compared to other cancers, the hope arising from clinical trial results is often challenged in the real-world setting, due to variability in patient demographics and clinical practice [11,12]. The simultaneous addition of several agents and various combinations of drugs with different intensities to clinical practice fuels research interest to assess the transferability of registrational trial data to the real world and to record the cumulative cost–benefit ratio versus the previous standard of care for healthcare providers.

To date and in contrast to other non-oncology therapeutic areas, there are no real-world RRMM studies investigating the cumulative comparative benefit of a “generation” of pharmaceutical innovation versus the prior standard of care and the impact that earlier adoption of novel drug combinations may have on patients and healthcare systems. The aim of this study was to evaluate the clinical outcomes attributed to the simultaneous introduction of several novel agents and real-world drug combinations in the management of RRMM in Greece over the recent years and to subsequently analyze findings in the context of baseline outcomes from the prior standard of care. Future analysis of findings is needed to compare the economic burden among past, current, and future therapeutic algorithms.

## 2. Materials and Methods

This was a national, retrospective, observational, multi-center chart review study in the relapse refractory multiple myeloma landscape in Greece. In order to evaluate the pharmacoepidemiological consequences associated with the simultaneous introduction of several therapeutic alternatives in real-world clinical practice, the study objectives focused on approved pharmaceuticals as per their European labels described in the respective summary of product characteristics. Three hospital sites participated, with satisfactory geographic distribution across mainland Greece (2 rural university hospitals and the largest tertiary public hospital in Greece).

The population of interest included patients with RRMM who had received 2nd- and/or 3rd-line MM treatment with carlizomib, daratumumab, elotuzumab, ixazomib, panobinostat, or pomalidomide as approved monotherapy or their combinations after European approval and up to the date of data extraction in 2023. Patients were selected based on pre-specified inclusion and exclusion criteria (Table 1). The approval cutoff for the selection of drugs to be studied in the research protocol was December 2019 and was based on the European Medicines Agency status. The decision to focus on treatment regimens that were approved more than 3 years prior to data extraction guaranteed adequate time for real-world data build-up and accounted for potential delays in public funding and patient access. Sequential treatment-based enrollment was followed up to the site target (30–90 subjects per site, depending on the primary investigator’s preliminary assessment of selection criteria) or until all subjects who met the selection criteria were enrolled to limit selection bias. As an observational study with a descriptive analysis plan, the estimated target per drug was not binding.

In cases where the same patient received two of the study drug agents in the 2nd and 3rd lines, respectively, this subject would count as a single entry and data were collected for both lines of therapy if selection criteria were followed.

The first primary endpoint, for both 2nd-and 3rd-line settings, was the 52-week PFS rate, defined as the proportion of patients who did not have a recorded treatment discontinuation, or had not initiated a subsequent line of therapy (in the absence of records of relapse or non-response), or were not recorded as deceased at any time prior to the completion of a 365-day period from the first dose. The other co-primary endpoint was the median PFS-2, defined as the sum of treatment lengths across the 1st and 2nd lines of therapy. All clinical endpoints are included in Table 2. The selection of primary endpoints was based on the results presented in similar real-world studies during the lenalidomide era in Greece [13,14] to ensure indirect comparability of clinical outcomes between the “before” and “after” of the simultaneous entry of several novel agents.

Other exploratory endpoints included direct costs associated with the management of multiple myeloma patients per year of therapy in the 2nd- and 3rd-line MM settings and the distance of the primary residence from the hematology center (for patients and caregivers, as available), alongside travel means used and other out-of-pocket expenses. Such data will support pharmacoeconomic analysis and will be presented in the future.

Due to the nature of this study, simple descriptive statistical analysis was performed wherever possible. The PFS rate at 52 weeks followed a binary “yes” or “no” classification at the 365-day cutoff. Percentage and mean/median values were estimated with 95% confidence intervals for the ORR and the time to best response. It was noted that the purpose of this study was not to compare individual study drug effectiveness in the real world, mainly due to sample size limitations. Our research question focused instead on the collective pharmacoepidemiological footprint, and analysis was stratified based on the line of MM therapy. In terms of the PFS, the statistical tool used for analysis was the Kaplan–Meier estimator, with 3 hypothesis tests (log-rank, Breslow, and Tarone–Ware) to compare the survival distributions of any 2 groups, if applicable (e.g., exploratory comparisons between drug classes only if adequate distribution as a percentage of the total number of enrolled patients in the 2nd line of therapy). Furthermore, the Kaplan–Meier estimator was used to calculate PFS probabilities from censored data. PFS curves were constructed by plotting the survival function against time. Sankey plots were used to better demonstrate the real-world treatment sequencing and the associated median duration of therapy from the 1st to 3rd LOTs. Odds ratios were compared in a forest plot to assess the influence of different disease and patient characteristics on the likelihood of maintaining the PFS at 52 weeks post-2nd LOT initiation. Patients were stratified by age (younger diagnosis <65 years or >65 years), ASCT eligibility (yes or no transplantation), ISS stage (III vs. I and II), time since MM diagnosis (>3 years), treatment refractoriness (double-refractory at 2nd LOT initiation), and quality of response in the 2nd LOT (PR). All statistical analyses were performed using SPSS v 24.0 software.

Despite all quality assurance efforts and good recording practices followed in this study, data could be missing completely at random. Missingness of data was assumed to exhibit no specific patterns in this study design and statistical methodology. In the case of missingness (<1% vs. total variable entries), the statistical analysis plan adopted replacement of any missing values with K-nearest neighbors from the dataset. In the case of categorical data missing, variables were replaced with the most frequent value.

A feasibility assessment was carried out prior to protocol finalization, due to the restrictive nature of the patient selection criteria. Site investigators were responsible for data collection from patients’ medical records using a paper CRF. Trained members of a clinical research organization (CRO) oversaw site initiation visits that included protocol, ICF, and CRF training. An in-person monitoring visit followed enrollment of the first 10–25 subjects per participating site. Data were transcribed onto the master electronic database, and missing data mapping was performed by the CRO. Further communication with sites was permitted to ensure good recording practices and/or reduction in cases with missing data, wherever possible.

## 3. Results

Data from 107 patients with RRMM were collected between March and August 2023 (first subject on 29 March and last subject on 1 August). Of the 107 database entries, 5 were excluded from analysis as they were found to deviate from the pre-specified inclusion and exclusion criteria (non-approved combinations and insufficient length of records). Another four subjects were found to have data with minor deviations to exclusion criterion number 2 (see Table 1). Specifically, in all four cases, one of the study drugs was received in the second line of therapy; however, the administered monotherapy or combination scheme was only part of an approved regimen or had dose variations outside the approved EU label, and hence, any results were presented separately. Of the 98 entries across the three research sites, 76 subjects received the study drug(s) as second-line MM treatment and 51 met the inclusion criteria for third-line MM treatment. A total of 27 subjects received the study drug as monotherapy or in approved combinations across second and third lines of MM treatment.

Based on recommendations from the primary site investigators, panobinostat and elotuzumab had not been prescribed in any of the hematology clinics that participated in the study and, therefore, were excluded from the clinical research form and analysis. Notably, the database only suffered a negligible number of entries with missing data and such gaps did not influence any outcome measures.

### 3.1. Patient Characteristics

Baseline characteristics at first relapse are presented in Table 3. The median patient follow-up from the date of RRMM diagnosis or, if missing, from second-line treatment initiation was 2.0 years (mean was 3.8 years).

### 3.2. Treatment Patterns and Treatment Duration

Data on first-line treatment after MM diagnosis were collected for all patients. The majority of patients received bortezomib- or lenalidomide-based regimens (62% and 18%) or a combination of bortezomib, lenalidomide, and dexamethasone (17%). 

In the second-line setting, 34% of patients received daratumumab-based schemes, 18% received carfilzomib-based schemes, 22% of patients received ixazomib–lenalidomide–dexamethasone therapy, and 16% of patients received pomalidomide-based schemes. Additionally, 5% of patients received a combination containing daratumumab and pomalidomide, whereas another 4% of patients received a combination of daratumumab and carfilzomib. Two of the subjects who were excluded due to minor regimen deviations received daratumumab monotherapy or a combination with dexamethasone, one received ixazomib–dexamethasone therapy, and one received pomalidomide–dexamethasone therapy in the second-line setting. A third of patients received lenalidomide, including combinations with pomalidomide or ixazomib or carfilzomib after having received lenalidomide in the front-line setting.

In the third-line setting, 37% of patients received daratumumab monotherapy or part of the approved combination, 29% received pomalidomide-based schemes, 8% received ixazomib–lenalidomide–dexamethasone therapy, 10% received carfilzomib-based regimens, 14% received a combination containing daratumumab and pomalidomide, and, finally, 2% were prescribed carfilzomib–daratumumab therapy. Of the 18% of subjects who were classified as double-refractory having progressed on a proteasome inhibitor and immunomodulatory agent in the front-line setting, eight subjects received a daratumumab combination in the second LOT and only one maintained progression-free survival at data extraction.

Treatment duration for enrolled subjects per line and cumulatively for two lines of therapy is presented next to the respective mean and median values and includes subjects who may have received the non-study drug in either the treatment at first relapse or in the third-line setting (Table 4). Interestingly, longer mean treatment durations were observed for both subgroups in the third-line treatment landscape when compared to first- and second-line settings. A further analysis of the subgroup of subjects who received study drug combinations or approved monotherapies in both second and third lines of therapy showed that the mean and median were 36 and 31 months, respectively, despite the 2-year median follow-up from the first RRMM record. 

**Figure 1:**
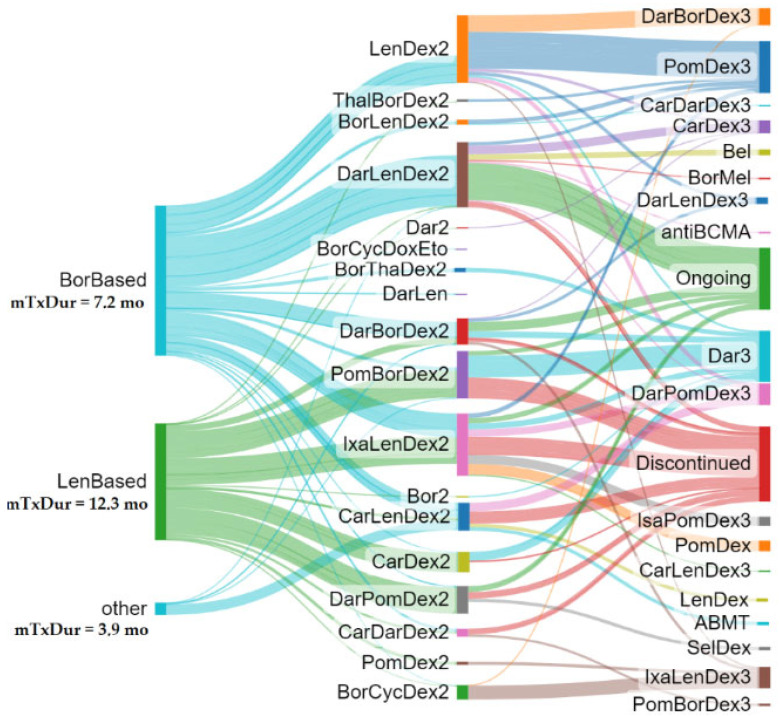
Treatment duration and sequence patterns. Created with SankeyMATIC. Subjects on the 2nd LOT who remained in the PFS at data extraction are shown as “ongoing” in the 3rd LOT. “Discontinued” stands for drug discontinuation due to any reason or lost to follow-up. 2nd LOT DarLenDex = 10.5 mm, IxaLenDex = 10 mm, and PomBorDex = 7.5 mm.

Figure 1 summarizes the treatment sequences for the total subjects. The Sankey plot tree was grown out of the first LOT selection, and treatment algorithms were followed up to the third LOT or discontinuation/loss to follow-up. Individually colored branch thickness is reflective of the median treatment duration to demonstrate how various therapeutic algorithms may have translated in real-world treatment durability (the wider the branch, the higher the treatment duration).

When focusing on patients who received a study drug combination in the second LOT, a higher treatment duration was observed for DarLenDex, IxaLenDex, and PomBorDex.

### 3.3. Progression-Free Survival

When removing data for four patients who reached CR prior to the completion of the first year on study drug therapy, the 52-week PFS rate in the second line of therapy was 72.2%. These patients could not be counted as having maintained the PFS at 52 weeks, since there was no confirmatory record available. Of the four subjects who received part of an approved scheme in the second-line treatment setting, only two showed PFS at 52 weeks. In the third-line treatment setting, the respective number for the PFS rate at 52 weeks was 63.5% (33 of 52 subjects; one subject had CR prior to 52 weeks and was excluded from the binary classification and the PFS rate calculation).

**Figure 2:**
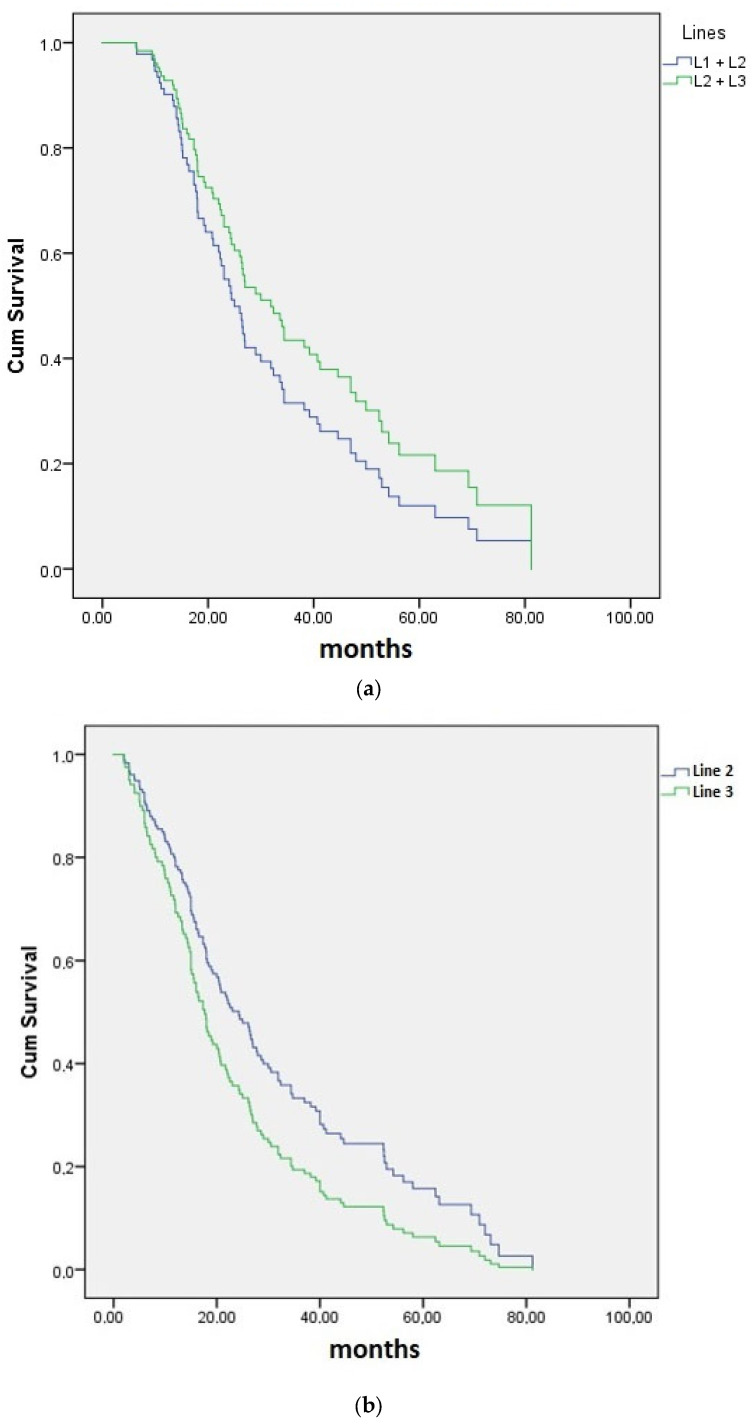

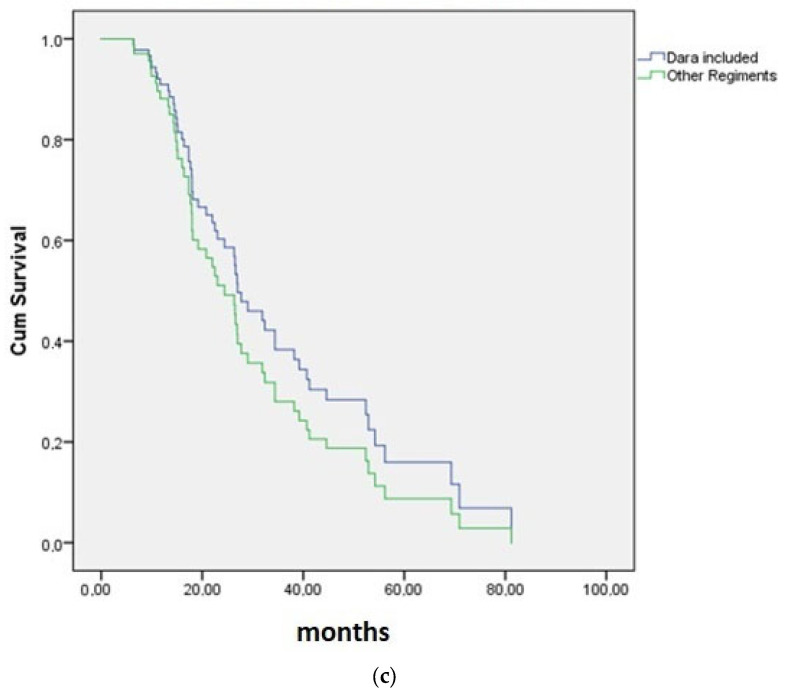
PFS curves per line of therapy or other subgroups in subjects who met the selection criteria. (**a**) PFS-2 (PFS line 1 + PFS line 2) for all subjects who received the study drug in the second line following any observed first line of therapy (N = 76) vs. PFS-3 for all subjects who received the study drug in both 2nd and 3rd line settings (N = 27). (**b**) PFS in 2nd vs. 3rd line of therapy (all study drugs). (**c**) PFS in daratumumab-containing regimens vs. all other study drug combinations in the second line.

The median PFS up to follow-up was 26.3 months (95% CI 21.3–31.3; mean was 31.9 months), 17 months (95% CI 13.3–19.7; mean was 25.5 months), and 15 months (95% CI 11.2–18.8; mean was 23.9 months), respectively, for PFS-2 (cumulative first and second lines), second, and third lines of therapy (Figure 1). The median PFS for the 27 subjects who received the study drug in both second- and third-line settings was 33.6 months (95% CI 20.6–46.0; mean was 39.2 months (Figure 1)). Observed differences between the median PFS in enrolled subjects who received any of the study drugs in the second and/or third line were not statistically significant in any of the PFS curves presented in Figure 2a,b.

Since more than a third of the subjects received daratumumab-based regimens at first relapse, the exploratory comparison of the median PFS between daratumumab-including regimens versus all other study drug combinations was deemed relevant and was found to be 34.4 (95% CI 16–53) versus 23 (95% CI 15–31) months, respectively. Statistical significance was not reached in all three statistical tests (chi-square Mantel–Cox *p* = 0.216, Breslow *p* = 0.190, and Tarone–Ware *p* = 0.204). However, a clear trend in favor of daratumumab-based schemes could be observed (Figure 2c).

**Figure 3:**
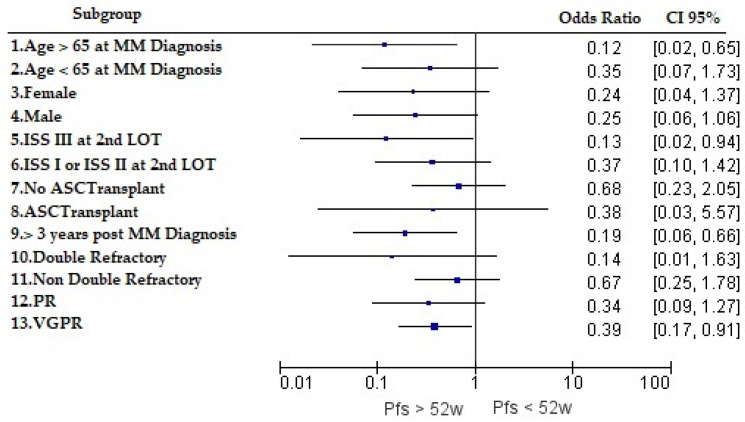
Influence of disease and patient characteristics on the 52-week PFS rate. Key: 10. “Double-refractory” defined as PI and immunomodulatory agent at 2nd LOT initiation; 11. “Non-double-refractory” defined as all other front-line treatments not including a combination of a PI and immunomodulatory agent; 12. “PR” as the best response in the 2nd LOT; 13. “VGPR” as the best response in the 2nd LOT.

The PFS rate at 52 weeks was stratified by disease or patient characteristics. Odds ratios (95% CI) were estimated and are presented for different patient subgroups in Figure 3. Age greater than 65 years at MM diagnosis, achieving VGPR, and having received a diagnosis and first-line treatment more than 3 years prior to data extraction seemed to positively influence the likelihood of achieving PFS at 52 weeks post-second LOT (statistical significance).

### 3.4. Response to Treatment

Objective response, defined as physician-reported ≥partial response (PR), was observed in 76.1% of enrolled subjects in the second line of treatment, whereas the respective percentage dropped to 69.7% in the third-line setting. Specifically, 43.8%, 17.7%, and 14.6% of subjects had VGPR, PR, or CR recorded, respectively. Progressive disease was recorded within a median of 6.4 months in approximately 3% of patients after initiating treatment at first relapse. In the third line of therapy, VGPR was reached in 35.8%, PR in 22.6%, and CR in 11.3% of subjects. Stable disease was observed in 20.8% of subjects in the second and 17% in the third line of therapy. Median and mean times to best response are presented in Table 5.

### 3.5. Reason for Study Drug Discontinuation in 2nd and 3rd Lines of Treatment

Non-response or progressive disease were listed as the most common reasons for drug discontinuation across both lines of therapy under evaluation (53% of subjects who discontinued in both second and third lines). The second-most identified reason for study drug discontinuation was related to the tolerability profile of the agent and the adverse events experienced by the patient. In both second- and third-line treatment settings, one-fifth of the patients were deprived of further treatment benefit due to the latter reason. Other reasons for study drug discontinuation are included in Table 6. Third-line data entries had proportionally more missing data compared to second-line datapoints, mainly in cases of ongoing therapy beyond data extraction.

## 4. Discussion

This is the first real-world study to evaluate the clinical benefits of the simultaneous entry of several novel pharmacotherapies in the management of RRMM in Greece. Despite the numerous interventional and observational studies contributing to the continuous assessment of clinical outcomes, this study, despite its limitations, offers a novel prototype methodology for indirect comparability of outcomes between different “generations” of pharmaceutical innovation. The findings of this study build upon previous work in the era of bortezomib and lenalidomide by providing updated insights into the effectiveness and tolerability of novel agents in the second-line setting and by adding new observations for subsequent lines of therapy in the real-world management of RRMM. 

The International Myeloma Working Group’s recommendations for the management of relapse refractory disease are based on lenalidomide refractoriness at first relapse. Another question that has been posed in the decision tree of this guideline is whether the disease has progressed on first-line therapy that includes daratumumab [9]. Our sample included approximately 25% of patients who had received lenalidomide either with dexamethasone or in a combination with bortezomib, and a negligible number of subjects in the overall cohort (N = 107) had received daratumumab in the first-line setting (<1%). In line with the international recommendations, most REAL IMM patients received daratumumab-based therapy at first relapse, since the majority of cases were not classified as refractory to lenalidomide (approx. 80% had received bortezomib in the first line of therapy). As observed in our study, alternative therapeutic approaches at first relapse also included a balanced proportion of prescribed carfilzomib-, ixazomib-, and pomalidomide-based schemes. The management of patients with RRMM who have received two or more prior lines of therapy remains challenging as more novel agents are used in the front-line setting or at first relapse [9]. The international recommendation is to reconsider all agents not used at first relapse or to enroll the patient in a clinical trial, wherever possible. Since most phase 3 clinical trials exclude lenalidomide refractory patients when investigating the efficacy and safety of triple combinations including lenalidomide in the second-line setting, the myeloma community does not currently have adequate data to understand the benefits of lenalidomide rechallenge as part of a combination following lenalidomide refractoriness in front-line MM settings. In our study, a third of the subjects who had received lenalidomide in the front-line setting were rechallenged or were switched to a lenalidomide-containing triple combination with carfilzomib, ixazomib, or pomalidomide at first relapse. A significant percentage of patients were double-refractory at second LOT initiation based on the IMWG 2016 consensus on the criteria for response and minimal residual disease and related double- and triple-class refractory definitions [15]. Approximately one in five subjects were refractory to a first-line combination of a proteasome inhibitor and an immunomodulatory agent, and only one of the subjects who was triple-class refractory having received daratumumab combination in later lines of therapy was still in PFS at the time of data extraction. All other patients classified as triple-class refractory experienced disease progression in later lines. This confirms prior findings about the challenging-to-treat triple-class refractory population [16].

Real-world evidence on carfilzomib–lenalidomide and dexamethasone therapies from Mele et al. [17] and Rochi et al. [18] found a similar median PFS and reported ORR at 79–88%. A small real-world study from Turkey showed that response to carfilzomib–dexamethasone therapy was lower than in clinical trials; however, the study had follow-up and sample size limitations [19]. Terpos et al. [20] found that the ORR in the real world was 83% and 68% and the median DOT was 14.6 months versus 7.5 months for Krd versus KD, respectively (N: Krd = 382, Kd = 273).

Small real-world studies on daratumumab monotherapy have demonstrated slightly lower effectiveness than clinical trials [21]. A large Canada-based registry study (N = 710) [22] confirmed the improved results of daratumumab when combined with lenalidomide instead of bortezomib. The median PFS for daratumumab–lenalidomide therapy was 26 months.

Several real-world studies are supportive of the ixazomib clinical trial findings [11,21]. Terpos et al. [23] (N = 155) enrolled heavily bortezomib- and carfilzomib-pre-treated patients (91%) on IRd and showed a real-world PFS of 27 months, whereas Cohen et al. showed a slightly better ORR (88%) and a median PFS of 24 months [24].

Based on systematic reviews [11,21], real-world effectiveness results for pomalidomide are in line with published clinical trials. In these reviews, the ORR ranges from 32 to 53% in pomalidomide real-world studies.

The depth of response and time to subsequent treatment decreased with each additional line of therapy in a much bigger European chart review study (N = 4997 MM patients) [25]. Researchers found that the proportion of patients achieving complete response (CR) decreased from 32% at the first line of therapy to 4% at the fourth line of therapy. Similarly, 74% of subjects achieved good partial response (VGPR) in the first-line setting compared to 11% at the fifth line of therapy or later. Furthermore, the median time to progression for patients who completed first-line treatment was 18 months. In the second line of therapy, the median time to progression was 13 months, with 10% of patients progressing after 36 months or more. The median time to progression for patients in the third line of therapy was 7 months. A significant contradiction emerges when comparing the findings of this pan-European study to the results of REAL IMM. Specifically, because of the innovation leaps observed in the past decade, the cumulative median PFS across second and third lines of therapy (PFS-3 for easiness) trended higher than the median PFS-2 (PFS in the first line plus PFS in the second line), as shown in Figure 2a, despite the presumably more challenging profiles of relapse and refractory patients in later lines. Such PFS-2 trends are expected to improve once newer agents become the mainstay of front-line real-world MM.

In Greece, Katodritou et al. (2014) [13] looked at the real-world objective response with lenalidomide–dexamethasone therapy at first relapse. The objective response (≥PR) was 77.4%. Additionally, the authors found that CR was observed in 20% of the population and that the quality of response was independent of previous lines of therapies with bortezomib or thalidomide. The median duration of response was found to be 34.4 months. Similar response rates and quality of response have been observed by several research groups across the globe in the previously naïve lenalidomide RRMM population, which is a true testament of lenalidomide’s revolutionary pharmacoepidemiological footprint on a population level in RRMM. When comparing response rates, our study showed similar results (76% in the second-line setting), with a slightly higher percentage of patients achieving CR (median of 24.0 and mean of 28.2%). This observation may be explained due to the improved efficacy data of newer agents. The same group (Katodritou et al., 2018) [14] investigated the real-world effectiveness of lenalidomide–dexamethasone therapy at first relapse in terms of the PFS rate at 52 weeks and the median PFS. Similar to our study, the majority of patients had received bortezomib-based therapies in the first-line setting, with approximately 25% of subjects having gone through ASCT. The overall median PFS and the 52-week PFS rate were found to be 19.2 months and 67.6%, respectively. Notably, the median PFS was 24 months for treatment at biochemical relapse, whereas the median PFS dropped to 13.2 months in cases where subsequent therapy was initiated at clinical relapse. Interestingly, when comparing the overall findings to the respective results of our study, with the newer carfilzomib-, daratumumab-, ixazomib-, and pomalidomide-based algorithms, a similar median PFS and 52-week PFS rate were observed (17 months and 67.6%, respectively). This finding could be attributed to the fact that more patients had previously received lenalidomide in the front-line setting. It has to be noted that DarLenDex had not yet been used as front-line therapy in our study. The median PFS-2 (cumulative PFS across first- and second-line settings) in the REAL IMM study was 26.3 months (mean of 31.9 months) and 15 months (mean of 23.9 months) in the third-line setting, respectively. Older age at diagnosis, achieving VGPR, ISS stage III at second LOT initiation, and having received an MM diagnosis (and front-line care) more than 3 years prior to data extraction seem to positively influence the likelihood of maintaining the PFS in the second-line setting. The statistical significance reached with ISS stage III is of interest and may be explained by more intensified treatment selection for the second LOT. Such analysis demonstrates the importance of achieving ≥VGPR in the second line of therapy as it seems to influence PFS durability when PR does not reach statistical significance.

Treatment duration in this study was almost identical between all subjects who received the second and third lines of therapy and received study drug combinations (18.2–19.0 months in the second line and 19.7–20.6 months in the third line of therapy). Subjects who met the selection criteria and received either carfilzomib-, daratumumab-, ixazomib-, or pomalidomide-based regimens had a similar treatment duration compared to all patients on combinations including or not including the study drug. In the second LOT, a higher treatment duration was observed for DarLenDex, IxaLenDex, and PomBorDex. In a retrospective electronic medical record study in the United States, Chari et al. identified 664 patients with RRMM who received 733 lines of therapy (IRd N = 168, Krd N = 208, VRd N = 357) [26]. The median follow-up for all patients from initiation of the index line of therapy was 14.4 months (lower than the 24 months in the REAL IMM study). Variations were observed as treatment duration was the longest for those treated with IRd (12.3 months) versus KRd (7.2 months) and VRd (10.0 months), with a significantly lower risk of IRd regimen discontinuation versus KRd. In any case, the overall median treatment duration in the second line of therapy or above for the entire regimen within the index LOT was shorter when compared to the findings of our study.

Davies et al. conducted a real-world comparative effectiveness analysis of bortezomib, carfilzomib, ixazomib, and daratumumab combined with either lenalidomide or pomalidomide plus dexamethasone in RRMM [27]. Patients were followed longitudinally until death, loss to follow-up, or end of the study period (from 1 January 2014 to 31 March 2018). Authors found that the unadjusted median time to next treatment in the second line of therapy or above was 13.9 for VRd, 8.7 for KRd, and 11.4 months for IRd. At the study follow-up, the median time to next treatment for DRd was not estimable. Figure 1 showed that the median treatment duration for DarLenDex was similar to that of IxaLenDex and higher than that of CarLenDex. Overall, it appears that the median treatment duration and the associated median PFS were notably longer in REAL IMM (mPFS second line = 17 mo, median treatment duration second line = 14 mo; mPFS third line = 15 mo, median treatment duration third line = 13.7 mo; and mPFS second plus third line = 33.6 mo, median treatment duration third line = 31.0 mo).

In the CONNECT-MM registry, it was reported that up to 40% of patients treated in standard clinical practice would be ineligible for enrollment to randomized controlled trials in newly diagnosed MM, due to eligibility criteria [28]. A safe conclusion based on several systematic reviews to date is the recognition of the efficacy–effectiveness gap between clinical trial results and outcomes of real-world studies [11,12,21]. Based on these systematic reviews, trials tend to include younger and presumably healthier patients. Other potential reasons for the gap between real-world effectiveness and clinical trial efficacy results may include the care setting effect (academic vs. community centers), study design, and physician or patient preference [29]. Patient selection based on such “sterile” criteria and the optimal management of toxicities would result in fewer patients requiring dose reductions and treatment discontinuations, ultimately introducing bias and outcomes’ inflation [30]. The aforementioned gap justifies investments in more real-world studies, which can in turn be used in decision making in clinical guideline and health technology assessments. Such a potentially impactful observational analysis was recently presented by Girvan et al. to retrospectively assess how different treatment regimens by LOT have evolved since the 2010s, who validated that real-world overall survival continued to drop with ascending LOTs [31]. Although not directly comparable to survival outcomes, the explanation as to why 2nd and 3rd LOTs in this study had cumulatively better mPFS rates when compared to 1st and 2nd LOTs, may be the time lag between pharmaceutical innovation entry between the U.S.A. and Eastern Europe.

As with all retrospective chart review designs, this study has certain quality and methodology limitations. The small sample size due to the limited number of participating sites remains the biggest challenge for such methodology, which could be in the future used for broader explorations across RRMM and other hematologic malignancies. The lack of genetic mutation data limited the association analysis between patient characteristics and outcomes. Although satisfactory homogeneity is assumed across the definitions used to describe staging of disease and other patient demographics in Greece, minor deviations may have been introduced due to clinical practice variations across the three participating sites. Selection bias cannot be excluded despite all efforts during protocol design and investigator training. Another inherent bias comes from potentially not including deceased subjects where family contact could not be established or was deemed inappropriate. The integrated analysis of pooled findings for all study-drug schemes did not permit conclusions for individual novel agents. In any case, the sample size would not be sufficient for subgroup analysis based on treatment. Cases with missing data were limited in this study design, despite the retrospective chart review strategy. Bigger longitudinal studies across several lines of therapies such as INSIGHT MM (e.g., 4th–5th LOT and penta-refractory populations) are needed as more agents enter the therapeutic arena [32].

## 5. Conclusions

This is the first real world study evaluating cumulative real-world effectiveness associated with the simultaneous entry of several therapeutic agents in the management of RRMM in Greece. It is of utmost importance to not only continue to monitor the discrepancies between real world studies and clinical trial results but also to act upon the efficacy—effectiveness gap with improved registrational study designs that will be easily generalizable to the real world. Findings of this chart review study demonstrated similar 52-week PFS and response rates compared to previously reported lenalidomide-based therapies in the second-line MM setting in Greece. The improved median PFS trends in later lines of MM therapy in comparison to earlier lines, despite the presumably more challenging patient profile, is encouraging and supports the need to continuously raise the innovation and evidence bars. While efforts continue to turn multiple myeloma into a curable hematological malignancy, all researchers ought to further study treatment patterns in later lines of therapies, beyond the front-line and second-line settings. Future cost-utility analyses will form the basis for drug evaluation in our modern world with scarce resources.

## Figures and Tables

**Table 1:** Inclusion and exclusion criteria.

Inclusion Criteria	
1.	Diagnosis of multiple myeloma
2.	Over 18 years of age (no upper limit)
3.	Relapse refractory patients with a recorded first-line multiple myeloma treatment
4.	Patients who have received rescue treatment for >1 cycle (including prior non-interventional study participation) and are receiving any of the study drugs in 2nd- or 3rd-line MM treatment as approved monotherapies or in approved combinations (as of 1st June 2022, by the EMA)
5.	Signed informed consent (patient) or 1st degree relative approval if patient deceased (consent waiver)
Exclusion Criteria	
1.	Patients who received any of the study drugs during a prior interventional clinical trial
2.	Patients who received any of the study drugs off-label as per EU indication(s). (Dose discrepancies are not an exclusion criterion if the patient receives a drug for an approved indication as part of an approved scheme.)
3.	Patients who received the study drug’s first dose less than a year prior to data extraction (excludes the 3rd line where the subject has received the 2nd-line first dose more than a year from data extraction—in such a case, the patient record will only include 2nd-line-related endpoints to maintain the study’s retrospective nature.)
4.	Patients who were administered the study drug during an interventional clinical trial or compassionate use program (any non-reimbursed administration) according to the treating physician’s testimony or patient records
5.	Patients who were administered any of the study drugs as 1st-line therapy when newly diagnosed, irrespective of eligibility for autologous transplant

Note: The research protocol specified that any of the study drugs could be excluded from the methodology as long as it had not been prescribed in clinical practice, following PI and sponsor alignment. Data for other novel agents that were approved later than the cutoff date but may have been used as part of an approved combination to the study drug, e.g., isatuximab, would be collected as “other drugs” in patients who met the selection criteria for at least one line of treatment.

**Table 2:** Main clinical endpoints.

**Primary**	
1.	52-week Progression-free survival (PFS) rate in the 2nd- and 3rd-line MM settings
2.	Median PFS-2 (cumulative 1st and 2nd lines)
**Secondary**	
1.	Median treatment duration per line of MM therapy (1st, 2nd, 3rd lines up to data extraction)
2.	Reason for treatment discontinuation (2nd–3rd lines of MM treatment)
3.	Objective response rate (ORR) in the 2nd line

**Table 3:** Patient characteristics.

Characteristics	
Gender (% male)	60.4
Mean (median) time since MM diagnosis	46.05 (24.07) months
Mean (median) age at data extraction excl. deceased	67.6 (69.0) years.
Percentage (%) of subjects that had ASCT	77.6
ISS categorization at second-line Tx initiation	
Stage I	34.0%
Stage II	29.2%
Stage III	36.8%
Percentage (%) of deceased patients at data extraction	11.2
Percentage (%) of double-refractory patients (proteasome inhibitor and immunomodulatory agent) in front-line setting	18
Mean number of lines of therapy at date of data extraction	2.58

**Table 4:** Treatment duration for different lines of therapy.

	Mean/Median (Months)
1st-Line setting—all subjects	14.4/8.5
2nd-Line setting—all subjects	19.0/14.0
2nd-Line setting—subjects who met selection criteria	18.2/14.0
3rd-Line setting—all subjects	19.7/13.2
3rd-Line setting—subjects who met selection criteria	20.6/13.7
2nd- + 3rd-Line setting—subjects who met selection criteria in both lines	35.9/31.0

**Table 5:** Time to best response per line of therapy.

	Median Time in Months (95% CI)	Mean Time in Months (95% CI)
2nd-Line MM Treatment Setting		
CR	24.0 (15–32)	28.2 (18–35)
VGPR	14.0 (11–18)	27.8 (18–35)
PR	18.0 (14–22)	24.3 (16–36)
3rd-Line MM treatment setting		
CR	16.0 (10.7–21.2)	29.0 (9.6–48.2)
VGPR	15.0 (6–20.6)	26.4 (14.2–38)
PR	30.5 (6.7–54.2)	34.5 (18.7–44.4)

**Table 6:** Reasons for treatment discontinuation.

	Non-Response or Disease Progression	Tolerability/Adverse Events	Death Due to Any Reason	Complete Response	Other Reasons	Unknown
	Percentage (%) of Enrolled Patients per Line of Therapy (Number of Patients)					
2nd Line	53% (31)	19% (11)	8% (5)	5% (4)	5% (4)	5% (4)
3rd Line	53% (19)	19% (7)	6% (2)	8% (3)	-	14% (5)

## Data Availability

The aggregate datasets analyzed in the current study are available from the corresponding authors upon reasonable scientific request. Restrictions may apply to the availability of these data and additional permissions are needed according to participating hospitals Evangelismos General Hospital, Alexandroupolis University Hospital and Larisa University Hospital operating procedures.
